# A Neuro-Influence Experiment to Evaluate the Persuasiveness of Pre-Exposure Prophylaxis Promotion Messages Among Men Who Have Sex With Men: Protocol for a Randomized Controlled Trial

**DOI:** 10.2196/52546

**Published:** 2023-12-06

**Authors:** Cui Yang, Dominick Reyes, Marcus Henry, Lonnie Walker, Meghan Moran, Allison Mathews, Kathleen Page, Carl Latkin, Joseph D Tucker, Ian McCulloh

**Affiliations:** 1 Rutgers School of Public Health Piscataway, NJ United States; 2 Community Researcher Baltimore, MD United States; 3 JOY Baltimore Baltimore, MD United States; 4 Johns Hopkins Bloomberg School of Public Health Baltimore, MD United States; 5 Community Expert Solutions, Inc Durham, NC United States; 6 Johns Hopkins University School of Medicine Baltimore, MD United States; 7 School of Medicine University of North Carolina at Chapel Hill Chapel Hill, NC United States; 8 Arrow Analytics, LLC Lithia, FL United States

**Keywords:** HIV, men who have sex with men, neuroimaging, persuasion, PrEP, pre-exposure prophylaxis

## Abstract

**Background:**

Pre-exposure prophylaxis (PrEP) is recommended by the US Centers for Disease Control and Prevention, but behavioral factors limit uptake, especially among men who have sex with men. A better understanding of how humans cognitively process information may inform health message development to promote PrEP uptake.

**Objective:**

This paper is informed by the neuroscience of persuasion and influence and describes the protocol of a neuro-influence experiment using functional near-infrared spectroscopy (fNIRS) to evaluate the persuasiveness of PrEP promotion messages among men who have sex with men in Baltimore, Maryland.

**Methods:**

We will conduct a randomized controlled trial using fNIRS to measure brain activation among 60 participants viewing PrEP promotion messages either developed through a crowdsourcing open contest implemented by the study team or developed with a traditional social marketing approach. We will evaluate the effectiveness of PrEP promotion messages by assessing brain activation in the regions associated with persuasion and changes in PrEP willingness, behavioral intention, initiation, and action between the 2 groups.

**Results:**

This study is funded by the National Institutes of Health (National Institute of Mental Health: R34MH116725). Participant recruitment and data collection were completed in October 2023. The first results are expected to be submitted for publication in 2024.

**Conclusions:**

In addition to providing insight into the effectiveness of PrEP promotion messages, this study will examine the feasibility, acceptability, and utility of neuroimaging techniques to evaluate PrEP promotion messages for high-risk men who have sex with men. The findings can also demonstrate the utility of fNIRS as a tool for preproduct testing of health campaigns and enable the public health community to deliver more effective messages to improve health outcomes.

**International Registered Report Identifier (IRRID):**

DERR1-10.2196/52546

## Introduction

### Background

Pre-exposure prophylaxis (PrEP) is a highly effective HIV prevention drug for decreasing new infections [1]. There has been an increased effort to identify effective health communication strategies for increasing awareness, acceptability, and uptake of PrEP among those most at risk for HIV transmission as an HIV primary prevention tool [2]. Pretesting the effectiveness of health communication materials before large-scale implementation is highly beneficial given the significant expenditures associated with the implementation of a large-scale campaign [3]. However, some research suggests that using traditional methods to select public health campaign messages, such as self-reports, may not always be the best way to predict population-level behavioral change [4,5].

A better understanding of how humans cognitively process information may inform the selection of persuasive messages that are more likely to change attitudes and behaviors. A core foundation of many persuasion theories is that a message must connect to and resonate with an individual’s preexisting beliefs, values, and identity. Social judgment theory [6], for example, posits that all individuals have latitudes of acceptance around different attitudes—that is, alternate points of view they are willing to accept. Messages that fall outside this zone, into an individual’s latitude of rejection, will elicit counterarguments and will be rejected. Cognitive dissonance theory [7] offers additional insight into this process: individuals will be more likely to adopt messages that are consonant with, or can be incorporated into, their existing beliefs, values, and identity, and more likely to reject messages that are dissonant. Thus, messages that are too discrepant may result in no effect, at best, and a boomerang effect, at worst.

Empirical data on neural correlates of persuasion suggest that neuroscience may be significantly more reliable than traditional methods for understanding persuasion and behavior change [5,8,9]. A systematic review identified 20 studies that provided evidence of persuasive processing and outcomes of health communication messages using neurocognitive measures across different health behaviors (eg, smoking, sun safety, and narcotic substances), message types (eg, text-based phrases, video, and audio), and cultural groups [10].

**Figure 1:**
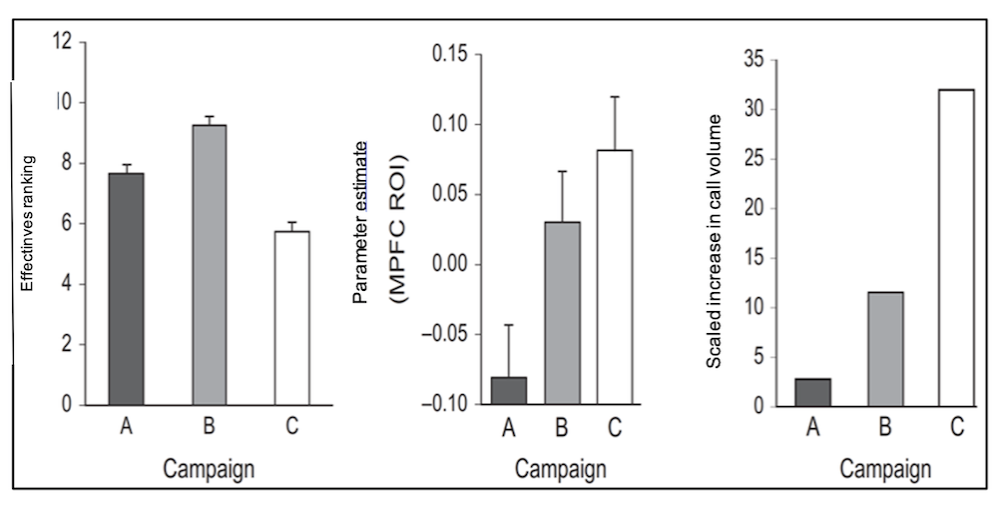
Comparison of self-report (left) and neural response (center) to tobacco cessation advertisements against effectiveness measure (right). MPFC: medial prefrontal cortex; ROI: region of interest.

The Neurocognitive Persuasion Model [11] identifies key brain regions associated with influence and persuasion. Accordingly, successful persuasion is associated with brain activity in the medial prefrontal cortex (MPFC) [6,12-14], through a process of self-integration, that is, increased brain activity for messages and cues that are successfully integrated with one’s self-concept [15]. In a population-level study [5], individuals viewed 3 tobacco cessation advertisements (A, B, and C) and were then asked to rank the effectiveness of the advertisements for helping them quit smoking on a scale of 1 to 10 (the left graph in Figure 1). The center graph in Figure 1 shows the average brain activity within the MPFC for those individuals viewing the advertisements, measured by functional magnetic resonance imaging (fMRI). To measure the population-level effectiveness of each advertisement, a 1-800 quitline call volume was assessed the month after each campaign aired. The right graph in Figure 1 shows the call volume after each advertisement was aired. Results indicated that the MPFC activity provides a stronger correlation to the real-world effectiveness of different advertising campaigns at the population level than self-report in this study, suggesting that neural response is a more reliable correlate of behavior change than self-reported perceived effectiveness.

In recent years, technological advances in the field of neuroscience have allowed us to study the structure of the brain and its functioning from a previously unknown approach. Functional near-infrared spectroscopy (fNIRS) is a noninvasive tool to measure key neural processes associated with influence and persuasion. fNIRS is particularly attractive for studying the neuroscience of persuasion and influence because most regions of the brain associated with this process are in the frontal cortex and accessible through fNIRS. The lower cost and greater portability of fNIRS make it a more cost-effective tool than fMRI, thus allowing scalability. In this study, we will use fNIRS to measure MPFC as the region most commonly predictive of individual- and population-level behavior change in past studies. There have been multiple studies that have used fNIRS measurements of MPFC to predict a person’s preferences [16,17]. To the best of our knowledge, there has been no published study using fNIRS in sexual health research or among highly marginalized populations.

### Objectives

Informed by the neuroscience of persuasion and influence, this paper aims to describe the protocol of a neuro-influence experiment using fNIRS to compare the effectiveness of 2 sets of PrEP promotion messages, one developed through a crowdsourcing open contest the study team implemented [18] and another developed through a traditional social marketing approach. Open contests, a form of crowdsourcing, involve a large number of community members in developing, vetting, and implementing solutions to public health problems in the form of a contest [19]. In our study, 79 PrEP promotion messages were submitted by community members, and then the top 4 entries were selected by 155 community votes in Baltimore. We expect the messages developed through open contests to be highly relevant to the community because open contests explicitly incorporate local knowledge, culture, and style by directly involving a large number of community members [20].

We hypothesize that individuals viewing PrEP promotion messages developed through crowdsourcing open contests will show higher brain activation in the MPFC regions than those viewing messages developed by a social marketing approach. We further hypothesize that brain activation in the MPFC regions will be significantly more correlated with PrEP behavioral intention, initiation, and action than self-reported message effectiveness.

## Methods

### Overview

We will conduct a randomized controlled trial using fNIRS to measure brain activation in MPFC among 60 participants. The key inclusion criteria for the participants are as follows: (1) 18 years or older, (2) biological male sex at birth, (3) never taken PrEP, and (4) being eligible for taking PrEP [21]. Participants will be recruited by community partners in Baltimore, Maryland, from a range of settings, including venue-based outreach (community-based organizations and health clinics) and word-of-mouth referral.

Participants will be randomly assigned to view 4 PrEP promotion messages developed through an open contest the study team implemented [18] or PrEP campaign messages developed with a traditional social marketing approach. We will compare brain activities between the 2 groups and follow up with participants in 30 days to assess any behavioral change.

### Study Procedure

After being screened eligible and providing written consent, each participant will be assigned a study ID and complete a baseline survey programmed in Qualtrics. Then we will make an appointment for an in-person experimental visit, roughly a week after completing the baseline survey. During the experimental visit, participants will be randomized to one of two conditions: (1) the top 4 messages developed through an open contest (ie, the intervention group) or (2) a total of 4 PrEP campaign messages developed with a traditional social marketing approach (ie, the control group). The participant’s assigned condition will be recorded in a logbook. Block random assignment (in batches of 10) will be used to keep the sizes of the 2 groups similar. The order in which the groups are assigned in each block is randomized and determined by a computer algorithm. This process is repeated for consecutive blocks of study participants until all participants are randomized.

After the randomization, a research assistant will take the participant to a private room. The research assistant will measure and fit the participant’s head for the fNIRS headband, which is connected to an NIRSport2 (NIRx) [22]. NIRSport2 is a portable fNIRS device to measure the neural response as participants view 4 messages on a computer screen. Once participants are fitted with the headband, the signal quality will be checked. The lights in the room will be turned off during testing to limit ambient light. A black shower cap will be placed over the participant’s head to further limit any potential ambient light, and the research assistant will verify signal quality again. The LED emitter and detector on the headband will measure brain activity when participants view messages. Participants in each group will view 4 messages programmed in PsychoPy [23] on a computer screen while brain activity will be recorded. Each message appears on the screen for 10 seconds, and there are 10 seconds of resting time between messages. Messages are ordered randomly, and we did not find any ordering effect in our previous study [11]. After reviewing all messages, participants will answer questions on self-efficacy (individual’s beliefs about their ability to follow through on a given behavior) and willingness and behavioral intention to use PrEP (see the “Measures” section). Semistructured questions on the acceptability of fNIRS will also be asked. All participants will complete a 2-week follow-up call to assess PrEP behavioral outcomes.

### Measures

#### Brain activity

**Figure 2:**
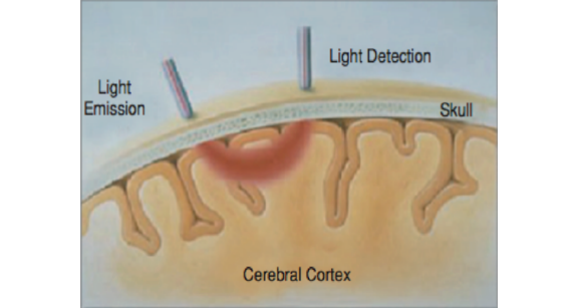
Functional near-infrared spectroscopy measurement of cerebral cortex.

fNIRS detects the ratio of oxygenated hemoglobin (O2HB) to deoxygenated hemoglobin (HHB) based on its optical properties, with higher ratios indicating greater neural activity. Neural tissues are relatively transparent to light in the near-infrared range between 700 nm and 1000 nm, and O2HB and HHB reflect distinct wavelengths in this range. By modulating light at different wavelengths, emitting the light on the skin-exposed areas of the cranium, and detecting that light, fNIRS measures the blood oxygenation level–dependent signal like fMRI. Because the photons reflected from neural tissue follow a reliable “banana-shaped” path, photodetectors can be used to measure this on the scalp (see Figure 2). By measuring the amount of absorbed light directed at the neural tissue, we can measure the amount of O2HB and HHB present in a region of the brain at a given time and draw inferences about the level of neural activity.

#### PrEP-Related Beliefs, Willingness, Behavioral Intention, and Behavior

All questions will be asked to participants at baseline (only to individuals aware of PrEP), at the second visit after reviewing all messages, and at the 30-day follow-up, except questions on PrEP action and initiation, which will only be asked at the 30-day follow-up. Self-efficacy [24] is assessed on a 5-point Likert scale ranging from “strongly disagree” to “strongly agree” to rate their agreement with the statement “I feel confident I could take PrEP as prescribed.” Willingness is assessed by asking participants, “Suppose that PrEP is at least 90% effective in preventing HIV when taken daily. How likely would you be to take PrEP if it were available for free?” with responses ranging from “I would definitely take it” to “I would definitely not take it” [25]. We will assess behavioral intentions to actually begin PrEP based on a real-world situation [25]. To do this, participants will be asked, “PrEP is currently available with a prescription from your doctor and research has shown that a majority of insurance companies cover most or all of the costs of PrEP. Do you plan to begin PrEP?” Response options range from “yes, I will definitely begin taking PrEP” to “no, I definitely will not begin taking PrEP.” Those participants who indicate they intend to take PrEP will also be asked a follow-up question regarding how soon they plan to begin taking PrEP. PrEP action and initiation will be assessed by asking, “Have you spoken with a provider about PrEP during the past 30 days?” and “Have you started taking PrEP during the past 30 days?” [25].

#### Message-Related Measures

All questions will be asked after participants finish reviewing each message during the second visit. Perceived effectiveness will be measured using a validated scale [26]. Participants will be asked, on a 5-point Likert scale ranging from “strongly disagree” to “strongly agree,” to rate their agreement that the message is believable, new, unconvincing, and important to them; helps them feel confident about using PrEP; would help their friends use PrEP; and puts thoughts in their mind about using PrEP and how much they agree or disagree with the message.

#### Acceptability of fNIRS

Participants will be asked to provide feedback on their experience with fNIRS, including questions: (1) “Do you feel comfortable wearing the cap?” with responses ranging from “extremely comfortable” to “not comfortable at all.” (2) “Do you feel the size of the cap is too small, a good size, or too big?” (3) “How do you feel about a machine reading your brain activity?” with responses ranging from “extremely comfortable” to “not comfortable at all.” (4) “How likely are you to participate in another study using the same type of machine in the future?” with responses ranging from “I would definitely take it” to “I would definitely not take it.”

#### Baseline Socioeconomic and Behavioral Factors

We will collect baseline data on age, the highest level of education attained, living situation, employment status, relationship status, biological sex at birth, gender, sexual identity, sources and amount of personal and household income, health insurance status, usual health care, and sexual behavior in the past 90 days.

### Data Analysis

We will conduct a descriptive analysis of the acceptability of fNIRS. The primary aim of data analyses is to assess the effectiveness of PrEP promotion messages developed through an open contest by assessing brain activation in the MPFC regions and changes in PrEP willingness, behavioral intention, initiation, and action between participants in the intervention group and those in the control group. We will compare (1) the level of neural activity in MPFC, (2) the increase in willingness and behavioral intention to take PrEP between baseline and 30-day follow-up, and (3) PrEP action and initiation at 30-day follow-up between participants in the 2 groups. A secondary aim is to evaluate the neural correlates of behavioral change.

Neural time series data will be captured for 23 brain regions in the prefrontal cortex. The time series signals will be averaged for each stimulus duration and for each 30-second rest period in between stimuli. These block averages will in turn be averaged across participants from the 2 groups. The level of brain activation for the intervention group compared with the control group will be evaluated using a pairwise comparison. For the binary outcome measures, that is, the increase in willingness and behavioral intention to take PrEP and PrEP action and initiation, we will first compare proportions by groups separately for each measurement period using standard bivariate analytic techniques such as Spearman rank-order correlations, odds ratios, and the Fisher exact test. Then we will use generalized linear mixed models (GLMM) to evaluate the effectiveness of messages developed through an open contest. GLMM permits us to measure the fixed effect of the messages developed through an open contest on neural activity, willingness, and behavioral intention to take PrEP, and PrEP action and initiation while allowing a random effect that accounts for within-person correlation. Given the binary nature of 2 primary outcomes, we will specify a logit link function for our GLMM models. Comparisons of the characteristics of participants who are lost to follow-up to those who are evaluated will be conducted to assess for systematic patterns that could influence results.

Self-reported effectiveness for individual messages will be averaged to compute self-reported message effectiveness for each group. We first examine the overall ordering of (1) self-reported message effectiveness by group assessed by mean ratings, (2) neural activity by group assessed by mean MPFC parameter estimate, and (3) PrEP action and initiation by group assessed by proportion of participants reporting taking PrEP action and initiation during 30-day follow-up. We next use a chi-square test to compare the proportion of individuals who produce each possible ranking with what would be expected by chance (1/6). Finally, we will confirm the reliability of the proportion-based predictions using weighted Kendall tau [27].

### Ethical Considerations

The study protocol has been approved by an institutional review board (Study ID: Pro2022000225). Screening for participant eligibility will be conducted in person or on the web after verbal consent. Once screened, eligible individuals will review the written consent and consent if they agree to participate in the study. Each participant will be assigned a study ID. Participants will receive US $15 for completing the baseline survey, US $60 for completing the experimental visit, and US $10 for completing the follow-up call.

## Results

This study is funded by the National Institutes of Health (National Institute of Mental Health: R34MH116725). Participant recruitment and data collection were completed in October 2023. The first results are expected to be submitted for publication in 2024.

## Discussion

Findings from the study will improve the scientific understanding of health communication in HIV prevention and treatment efforts. PrEP promotion messages identified as most persuasive with fNIRS will serve as a template for future contests with a focus on visual images (eg, color photographs, black-and-white photographs, and videos less than 1 minute) and distribution networks (eg, men who have sex with men social media networks, in-person men who have sex with men businesses, and in-person men who have sex with men social networks). The findings can also demonstrate the utility of fNIRS as a tool for preproduct testing of health campaigns and enable the public health community to deliver more effective messages to improve health outcomes.

We are well aware of the limitations of relying exclusively on small-scale pilots to determine whether messages developed through an open contest approach are more persuasive, namely that sizable standard errors are associated with effect sizes due to the small sample size. However, we are primarily interested in exploring the pattern of results for any evidence of support for the effectiveness of PrEP messages developed through the open contest on the primary outcomes. Effect size estimates determined through this study will be essential in the design of a larger and fully powered efficacy study that tests intervention effects on PrEP uptake.

In summary, the findings of this proposed study will transform the design, evaluation, and implementation of HIV campaigns, potentially bringing new ideas for local health departments and community-based organizations to develop more impactful PrEP campaigns.

## Appendix

Multimedia Appendix 1 Peer-review report by the National Institutes of Health, USA.

## Abbreviations

fMRI: functional magnetic resonance imaging
fNIRS: functional near-infrared spectroscopy
GLMM: generalized linear mixed models
HHB: deoxygenated hemoglobin
MPFC: medial prefrontal cortex
O2HB: oxygenated hemoglobin
PrEP: pre-exposure prophylaxis

## Data Availability

Data sharing is not applicable to this article as no data sets were analyzed during this study.
